# MEDIASTinal staging of non-small cell lung cancer by endobronchial and endoscopic ultrasonography with or without additional surgical mediastinoscopy (MEDIASTrial): a statistical analysis plan

**DOI:** 10.1186/s13063-021-05127-6

**Published:** 2021-02-27

**Authors:** Jelle E. Bousema, Jouke T. Annema, Erik H. F. M. van der Heijden, Ad F. T. M. Verhagen, Marcel G. W. Dijkgraaf, Frank J. C. van den Broek, on behalf of the MEDIASTrial study group, Nicole E. Papen-Botterhuis, Maggy Youssef-El Soud, Wim J. van Boven, Johannes M.A. Daniels, David J. Heineman, Harmen R. Zandbergen, Pepijn Brocken, Thirza Horn, Willem H. Steup, Jerry Braun, Rajen S. R. S. Ramai, Naomi Beck, Nicole P. Barlo, Martijn van Dorp, W. Hermien Schreurs, Anne-Marie C. Dingemans, Jos G. Maessen, Niels J. M. Claessens, Jan-Willem H. P. Lardenoije, Birgitta I. Hiddinga, Caroline van de Wauwer, Anthonie J. van der Wekken, Wessel E. Hanselaar, Robert Th J Kortekaas, Herman Rijna, Gerben P. Bootsma, Yvonne L. J. Vissers, Eelco J. Veen, Cor H. van der Leest, Emanuel Citgez, Eino B. van Duyn, Geertruid M. H. Marres, Eric R. van Thiel, Paul E. van Schil, Jan P. van Meerbeeck, Niels Smakman, Femke van der Meer, Mohammed D. Saboerali, Anne Marie Bosch, Wouter K. de Jong, Charles C. van Rossem, W. Johan Lie, Ewout A. Kouwenhoven, A. Jeske Staal- van den Brekel, Nike M. Hanneman, Roxane Heller-Baan, Valentin J. J. M. Noyez

**Affiliations:** 1 Department of Surgery, Máxima MC, Veldhoven, PO BOX 7777, 5500 MB Veldhoven, The Netherlands; 2 Department of Respiratory Medicine, Amsterdam University Medical Centre, University of Amsterdam, PO BOX 22700, 1100 DE Amsterdam, The Netherlands; 3 Department of Pulmonary Medicine, Radboud University Medical Centre, PO BOX 9101, 6500 HB Nijmegen, The Netherlands; 4 Department of Cardiothoracic Surgery, Radboud University Medical Centre, PO BOX 9101, 6500 HB Nijmegen, The Netherlands; 5 Department of Epidemiology and Data Science, Amsterdam University Medical Centre, University of Amsterdam, PO BOX 22700, 1100 DE Amsterdam, The Netherlands

**Keywords:** Non-small cell lung carcinoma, Mediastinal nodal staging, Endosonography, Mediastinoscopy, Thoracic surgery, Statistical analysis plan

## Abstract

**Background:**

Invasive mediastinal nodal staging is recommended by guidelines in selected patients with resectable non-small cell lung cancer (NSCLC). Endosonography is recommended as initial staging technique, followed by confirmatory mediastinoscopy in case of negative N2 or N3 cytology after endosonography. Confirmatory mediastinoscopy however is under debate owing its limited additional diagnostic value, its associated morbidity and its delay in the start of lung cancer treatment. The MEDIASTrial examines whether confirmatory mediastinoscopy can be safely omitted after negative endosonography in mediastinal nodal staging of NSCLC. The present work is the proposed statistical analysis plan of the clinical consequences of omitting mediastinoscopy, which is submitted before closure of the MEDIASTrial and before knowledge of any results was done to enhance transparency of scientific behaviour.

**Methods:**

The primary outcome measure of this non-inferiority trial will be unforeseen N2 disease resulting from lobe-specific mediastinal lymph node dissection. For non-inferiority, the upper limit of the 95% confidence interval of the unforeseen N2 rate in the group without mediastinoscopy should not exceed 14.3% in order to probably have no negative impact on survival. Since this is a non-inferiority trial, both an intention to treat (ITT) and a per protocol (PP) analyses will be done. The ITT and the PP analyses should both indicate non-inferiority before the diagnostic strategy omitting mediastinoscopy will be interpreted as non-inferior to the strategy with mediastinoscopy. Secondary outcome measures include 30-day major morbidity and mortality, the total number of days of hospital care, overall and disease free 2-year survival, generic and disease-specific health related quality of life and cost-effectiveness and cost-utility of staging strategies with and without mediastinoscopy.

**Discussion:**

The MEDIASTrial will determine if confirmatory mediastinoscopy can be omitted after tumour negative systematic endosonography in invasive mediastinal staging of patients with resectable NSCLC.

**Trial registration:**

Netherlands Trial Register NL6344/NTR6528. Registered on 2017 July 06

**Supplementary Information:**

The online version contains supplementary material available at 10.1186/s13063-021-05127-6.

## Background

Mediastinal nodal staging of non-small cell lung cancer (NSCLC) is important to determine treatment and prognosis. The European guidelines recommend invasive staging in patients with suspicious hilar or mediastinal lymph nodes on imaging (cN1-3) or centrally located, FDG-non-avid or large (> 3 cm) peripherally located tumours [1, 2]. Endosonography is recommended over surgical staging as initial staging technique. In case of tumour negative endosonography findings (no malignant N2 or N3 cytology) confirmatory mediastinoscopy is recommended in patients with cN1-3 and should be considered in patients with centrally located, FDG-non-avid or peripheral tumours > 3 cm to rule out false negative endosonography [1]. The use of confirmatory mediastinoscopy however is under debate owing its limited additional diagnostic value (number needed to test of 11), its associated complications (6.0%) or mortality and its delay in the start of definite treatment [3, 4]. The MEDIASTrial examines whether mediastinoscopy can be safely omitted after negative endosonography in invasive mediastinal nodal staging of NSCLC, based on non-inferiority [5]. The present work is the proposed statistical analysis plan (SAP) of the clinical consequences of omitting mediastinoscopy, which will be published before closure of the MEDIASTrial and before outcome measure data were available.

### Summary study protocol

The MEDIASTrial (MEDIASTinal staging of non-small cell lung cancer by endobronchial and endoscopic ultrasonography with or without additional surgical mediastinoscopy) is a multicentre randomised, parallel-arm, non-inferiority study in 342 patients with proven or suspected NSCLC. The complete study protocol was already published open access [5]. The hypothesis was ‘Omitting mediastinoscopy after negative endosonography in mediastinal staging of NSCLC does not result in an unacceptable percentage of unforeseen N2 disease at surgical resection (pN2). In addition, omitting mediastinoscopy will shorten time until definitive surgery, will prevent one general anaesthesia and hospital admission and will be associated with lower morbidity and comparable survival. Therefore, this strategy may increase quality of life and reduce health care costs.’

Patients with proven or suspected, resectable (judged by the thoracic surgeon on available imaging) NSCLC without distant metastases and with an indication for invasive mediastinal staging (i.e. cN1-3 or centrally located, FDG-non-avid or large (> 3 cm) peripherally located tumour) were eligible for inclusion. Prior to inclusion systematic endosonography with tissue sampling was performed (if indicated), resulting in tumour negative findings (no malignant N2 or N3 lymph nodes).

Patients with suspected metastases to lymph node stations 5 and 6 (i.e. aortopulmonary window) on imaging were eligible for inclusion. If metastatic spread to these nodal stations would lead to changes in treatment strategy according to the local multidisciplinary board extended invasive staging (i.e. parasternal mediastinotomy/scopy or VATS) should have been performed. In patients randomised in the group with mediastinoscopy, the regular cervical mediastinoscopy should have been expanded to investigate lymph node stations 5 and 6. Patients randomised in the group without confirmatory mediastinoscopy additional staging of stations 5 and 6 should have been done in a separate session or by using intra-operative frozen section analysis prior to the anatomic lung resection. If metastatic spread to station 5 or 6 would not influence treatment, patients were treated as described by the study protocol with or without confirmatory mediastinoscopy depending on randomisation outcome.

Exclusion criteria were ‘bulky N2-N3 disease’ on FDG-PET/CT, the combination of highly suspicious as well as irresectable mediastinal lymph nodes, non-correctable coagulopathy or insufficient comprehension of the Dutch language.

After inclusion, patients were 1:1 randomised to undergo either mediastinal staging with or without confirmatory mediastinoscopy. Randomisation was stratified by type of centre (Dutch academic, Dutch non-academic, Belgian academic) and by age up to or above 66 years. Patients assigned to staging with confirmatory mediastinoscopy received usual care conform existing guidelines. When histopathology after mediastinoscopy did not demonstrate N2 or N3 lymph node metastases, patients were recommended to undergo an anatomic resection of the primary tumour including lobe-specific lymph node dissection. Patients in the intervention-arm of the MEDIASTrial underwent immediate anatomic resection of the primary tumour including lobe-specific lymph node dissection without confirmatory mediastinoscopy.

The primary outcome measure for non-inferiority is the proportion unforeseen N2 disease, which is defined as pathologically proven N2 disease resulting from lobe-specific mediastinal lymph node dissection at time of tumour resection when previous invasive mediastinal nodal staging showed N0 or N1 disease. The pathological N stage results from the pathology report after pathological investigation, which was standardised by ‘The nationwide network and registry of histo- and cytopathology in the Netherlands’ [6]. Isolated cancer cell and micro-metastases were classified as positive findings when detected in lymph node dissection specimens.

Secondary endpoints include major morbidity and 30-day mortality, the total number of days of hospital care during 2-year follow-up, overall 2-year survival and generic and disease-specific health related quality of life. Additionally, a cost-effectiveness and cost-utility analysis of mediastinal staging strategies with and without mediastinoscopy will be done; this health economic perspective will be reported separately and falls beyond the scope of this analysis plan for assessing the clinical consequences.

The sample size calculation resulted in 171 patients to include in each randomisation group, or 342 patients in total (power 80%, alpha error 0.025). Based on an assumed 5% drop-out rate of patients after randomisation, we aim to include a total of 360 patients [5].

The medical ethical committee of Máxima Medical Centre approved the study protocol on June 15, 2017. The trial was registered at the Netherlands Trial Register on July 6, 2017 (NL6344/NTR6528). MEDIASTrial study protocol version 7.0, approved on July 1, 2019, is the latest and currently effective study protocol. The first patient was included on July 17, 2017, and the inclusion is expected to be complete in 2020. The full sample size calculation, study procedures and further details are available in the previously published trial protocol [5].

### Statistical analysis plan

The statistical analysis plan was conducted according to the Guidelines for the Content of Statistical Analysis Plans in Clinical Trials [7]. The checklist was provided in Additional file 1. FvdB is the clinical chief investigator and MD is the responsible senior statistician of the MEDIASTrial.

#### General principles

The primary analyses (for evaluation of primary outcome measure and major morbidity and 30-day mortality) will be performed when all patients have at least 30 days after the start of the treatment follow-up. The remaining secondary outcome measures will be analysed after completion of 2 years follow-up of all evaluable patients. Before analysing, the database will be cleaned and locked. No interim analysis will be performed. Analyses will be performed using the Statistical Package for Social Sciences (IBM SPSS Statistics for Windows, Armonk, NY). Generally, numerical outcomes will be presented as means (with standard deviation (SD) and/or range) or medians (with interquartile range (IQR and/or range)) depending on (normally or skewed) distribution of data. Numerical outcomes will be compared between groups using the unpaired *t* test or Mann-Whitney *U* test depending on distribution of data. Categorical data will be presented as counts and percentages and will be compared between groups using the Mantel-Haenszel chi-squared test or using Fisher’s exact test in case of zero cell counts [8, 9]. We will calculate 95% CI’s around proportions by using the Wilson score interval for proportions [10]. Correction for multiple testing of the secondary outcome measures will be done using the Benjamini-Hochberg method [11]. Statistical significance will be set at a *p* value of less than 0.05. In case data presentation or analysis is planned to be different, this will be stated in the specific outcome measure description part of this SAP. An overview of the planned statistical test per outcome measure to compare the randomisation groups is provided in Additional file 2.

#### Patient flow diagram

**Fig. 1:**
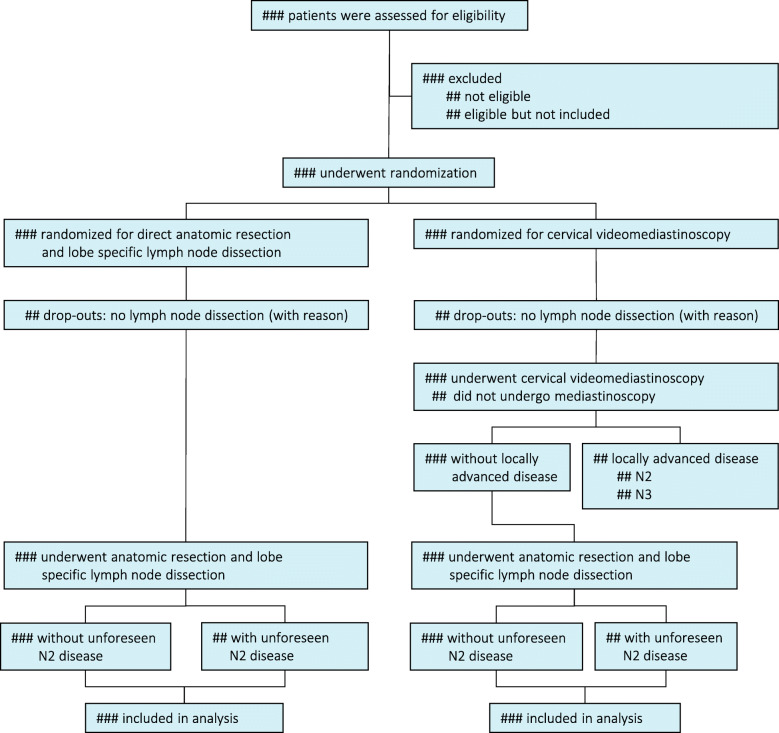
Enrollment, randomisation and flow of study patients. N2 = ipsilateral mediastinal lymph node metastasis; N3 = contralateral lymph node metastasis; Unforeseen N2 disease Pathologically proven N2 disease at lobe-specific lymph node dissection at time of tumour resection when previous mediastinal staging showed N0 or N1 disease

As indicated in the Consolidated Standards of Reporting Trials 2010 statement (CONSORT), the patient flow will be illustrated in a flow diagram (Fig. 1) [12].

#### Intention to treat and per protocol analysis

As this is a non-inferiority trial, both intention to treat (ITT) and per protocol (PP) analyses will be done [13]. The ITT and the PP analyses should both indicate non-inferiority before the diagnostic strategy ‘omitting mediastinoscopy’ will be interpreted as non-inferior to the strategy with mediastinoscopy. The pathology report of the lobe-specific lymph node dissection determines the nodal state, which is the primary outcome measure of this study. All patients from the ITT population without protocol deviations or violations in eligibility and study procedures will be included in the PP analysis. All analyses of secondary outcomes will be carried out on an ITT basis.

#### Protocol deviation and violation

Clinical deterioration and progression of the disease between randomisation and surgery could restrain surgical options and resectability of the primary tumour and lymph nodes. Patients in whom no lobe-specific lymph dissection was performed will be considered drop-outs since the primary outcome measures are missing. This population is expected not to exceed 5% as included in the sample size calculation. Patients randomised to confirmatory mediastinoscopy in whom no mediastinoscopy was performed prior to anatomical lung resection will primarily be analysed based on intention-to-treat. In per protocol analysis, these patients will be excluded for analysis.

#### Patient replacement and missing data

A 5% drop-out rate was included in the sample size calculation. As we assume the group of patients with missing primary outcome measures will not transcend this number, no patient replacement will be performed after inclusion of 360 patients. Clinical data management is done by professional data managers from the Dutch Comprehensive Cancer Centre. Any missing clinical data will be communicated to the study site data manager for prompt correction. Missing data in baseline characteristics (including FDG-PET/CT and endosonography results), mediastinoscopy, anatomic resection and lymph node dissection will not be imputed. For dichotomous variables, the actual denominator and for continuous variables the number of patients will be stated.

Randomisation outcome and treatment results (physical condition, complications, adjuvant therapy and oncological/survival results) could affect the number of completed questionnaires. Complete case analysis will be used as primary analysis for an outcome if the proportion of missing data is below 6% or missing data can be handled with mixed models or generalised estimation equations for repeated measures. In both instances, at least 342 evaluable patients should remain.

If less than 342 evaluable patients remain, missing data patterns will be studied to assess the likelihood of data being missing (completely) at random. Logistic regression on missingness of data will be applied to identify potentially associated baseline and clinical characteristics (e.g. gender, ASA-classification, indication for mediastinal nodal staging, clinical node stage, primary tumour location) and derive propensity scores for having missing data [14]. Subsequently, multiple imputation (*n* = 5) will be applied, including the propensity score, treatment allocation, type of centre, age at baseline, randomisation and stratification factors. Additionally the pathological results (pN stage), use of adjuvant therapy and the results of previously conducted questionnaires will be included. Alternatively, single imputation by ‘last observation carried forward’ replacing missing data with the last reported value of the same patient will be performed. Finally, a complete case analysis of available cases (*n* < 342) will be performed. Depending on the robustness of analysis results, a definitive choice for the method of handling missing data will be made. The imputation method with the smallest confidence interval and point estimates closest to the results of the complete case analysis of available cases will be considered the most robust one. In case of a lack of robustness because of changes in direction of the difference between treatment groups, worst and best case scenarios of imputation will be constructed. The handling of missing data will be extensively and transparently reported in supplemental material to the final results section.

#### Baseline characteristics

The following baseline characteristics will be reported in the baseline characteristics table: age, gender, type of centre, World Health Organization (WHO) performance state, American Society of Anesthesiologists (ASA) classification, primary tumour location (lobe), tumour and nodal stage according to the 8th TNM classification based on FDG-PET/CT, indication for invasive mediastinal nodal staging and the final histopathology result (Table 1). Testing for differences in baseline characteristics among groups will only be done if visual inspection of the results indicates possible significant differences.

**Table 1:** Clinical and lung cancer characteristics of included patients

	With mediastinoscopy (***n***=)	Without mediastinoscopy (***n***=)
Age, mean (SD)/median (IQR), y		
Sex, No. (%)		
Male		
Female		
WHO performance state, No. (%)		
WHO 0		
WHO 1		
WHO 2		
WHO 3		
WHO 4		
ASA classification, No. (%)		
ASA-1		
ASA-2		
ASA-3		
ASA-4		
Tumour location, No. (%)		
Left lower lobe		
Left upper lobe		
Right lower lobe		
Right middle lobe		
Right upper lobe		
Tumour stage FDG-PET/CT, No. (%)		
T		
Nodal stage FDG-PET/CT, No. (%)		
N		
Indication for invasive mediastinal nodal staging, No. (%)		
Clinical N1-3		
Central tumour		
FDG-non-avid tumour		
Peripheral tumour > 3 cm		
Final histopathology, No. (%)		
NSCLC		
Subtype		
Small cell carcinoma		
Benign		

*SD* standard deviation, *y* years, *No.* number, *WHO* World Health Organization, *ASA* American Society of Anesthesiologists, *FDG-PET* fluorodeoxyglucose positron emission tomography, *CT* computed tomography, *TNM* tumour, node, metastasis, 8th edition; *NSCLC* non-small cell lung cancer

#### Endosonography results

All included patients underwent systematic Endobronchial Ultrasound-guided Transbronchial Needle Aspiration (EBUS-TBNA), preferably with added Endoscopic Ultrasound-guided Fine Needle Aspiration by using the conventional endoscope (EUS-FNA) or the EBUS endoscope (EUS-B-FNA). We will report the following: the number of additional EUS procedures, sedation used, the proportion of procedures with rapid on site evaluation (ROSE), the number of visualised and sampled lymph nodes, the number of samples per lymph node station and the number of patients with cytologically proven N1 disease. The outcomes will be compared among the randomisation groups with subsequent presentation of outcomes for both individual groups (Table 2).

**Table 2:** Performance of diagnostic and therapeutic procedures

	With mediastinoscopy (***n***=)	Without mediastinoscopy (***n***=)
EBUS, No. (%)		
Additional EUS		
EUS, No. (%)		
EUS-B, No. (%)		
Rapid on-site evaluation, No. (%)		
Mediastinal lymph node stations		
Visualised, mean (SD)/median (IQR)		
Sampled, mean (SD)/median (IQR)		
Samples per station, mean (SD)/median (IQR)		
Cytologically proven N1 disease, No. (%)		
Confirmatory mediastinoscopy, No. (%)		0
Mediastinal lymph node stations		–
Sampled, mean (SD)/median (IQR)		
Adequate sampling^a^, %		–
Proven mediastinal lymph node metastases		–
N2, No (%)		
N3, No. (%)		–
Complete mediastinoscopy^b^, No. (%)		
Anatomical lung resection, No. (%)		
Thoracoscopic surgery, No. (%)		
Conversion to thoracotomy, No. (%)		
Duration of surgery, mean (SD)/median (IQR) minutes		
Resection type		
Segmentectomy, No. (%)		
Lobectomy, No. (%)		
Bilobectomy, No. (%)		
Pneumonectomy, No. (%)		
Mediastinal lymph node stations dissected, mean (SD)/median (IQR)		
Complete lobe-specific lymph node dissection^b^, No. (%)		
Unforeseen N2, No. (%)		
Foreseen N2 (station 5–6), No. (%)		

*EBUS* endobronchial ultrasonography, *EUS* endoscopic ultrasonography, *EUS-B* endoscopic ultrasonography using the EBUS bronchoscope, *No.* number, *SD* standard deviation, *N1* ipsilateral hilar lymph node metastasis, *N2* ipsilateral mediastinal lymph node metastasis, *N3* contralateral lymph node metastasis

^a^Adequate sampling = at least 4 surgical biopsies or one entire lymph node per station

^b^Complete according to the study protocol [5]

#### Cervical videomediastinoscopy results

We will report the number of visualised and sampled lymph node stations, the proportion of lymph node stations that were adequately sampled (i.e. at least four surgical biopsies or one entire lymph node) and the number of complete performed mediastinoscopy procedures (according to the study protocol) [5]. Additionally, the pathology results whether mediastinal lymph node metastases were found including the level of the affected lymph node stations will be reported (Table 2). A calculation of the number needed to test to detect a patient with missed mediastinal lymph node metastases after endosonography by performing confirmatory cervical videomediastinoscopy will be done. Complications of mediastinoscopy will be reported in the major morbidity and mortality outcome measure. The patients randomised for mediastinoscopy who did not undergo mediastinoscopy will be reported including the reason for this protocol deviation, if applicable.

#### Surgical reference standard

We will report the used surgical technique (video-assisted thoracoscopic surgery (VATS) single- or multi-port, thoracotomy), number of converted operations, duration of surgery (minutes), used type of resection (segmentectomy, lobectomy, bilobectomy, pneumonectomy), number of sampled mediastinal lymph node stations and the number of complete lobe-specific lymph node dissections (according to the study protocol) [5]. The outcomes will be compared among randomisation groups with subsequent presentation of outcomes for both individual groups (Table 2). Complications of the surgical lung tumour resection will be reported in the major morbidity and mortality outcome measure. The patients who did not undergo anatomic resection and lobe-specific lymph node dissection will be reported including the reason for not performing this procedure, if applicable.

#### Assessment and analysis of unforeseen N2 disease

Unforeseen N2 disease is defined as pathologically proven N2 disease resulting from lobe-specific lymph node dissection at time of tumour resection, not detected by invasive clinical staging including endosonography nor by mediastinoscopy (if performed). Patients with suspected stations 5 and 6 metastases on imaging who turned out to have pathologically proven station 5 or 6 metastases resulting from lymph node dissection will only be included in the unforeseen N2 calculation if pre-operative extended staging was performed (conform study protocol). In patients with suspect stations 5 and 6 on imaging in whom no extended staging was performed, pathological positivity of these nodal stations will be considered foreseen N2 disease, and thus not included in unforeseen N2 calculation. Patients with unsuspicious lymph nodes in stations 5 and 6 on imaging with pathologically proven metastases in these stations will be included in the unforeseen N2 calculation.

As substantiated in our study protocol, the upper limit of the two-sided 95% confidence interval (95% CI) of the unforeseen N2 rate in the intervention group (endosonography without mediastinoscopy) should not exceed the non-inferiority boundary of 14.3% in order to probably have no negative impact on survival [15]. A formal comparison of the unforeseen N2 rates of the randomisation groups with and without mediastinoscopy will be done based on intention-to-treat and per protocol analysis. Exploratory subgroup analysis of unforeseen N2 disease of patients with different indications for invasive staging (i.e. cN1-3 or centrally located, FDG-non-avid or large (> 3 cm) peripherally located tumour) will be performed.

Finally, an overview of all patients with unforeseen N2 disease will be provided. Unforeseen N2 disease will either be classified as detection error (lymph node metastasis not detected by FDG-PET/CT, endosonography nor mediastinoscopy) or sampling error (metastasis detected by FDG-PET/CT, but missed despite lymph node sampling during endosonography and/or mediastinoscopy).

#### Major morbidity and 30-day mortality

Complications in the first 30 days after start of treatment are scored using the Clavien-Dindo classification [16]. Major morbidity is defined as Clavien-Dindo grade III (requiring surgical, endoscopic or radiological intervention) or IV (life-threatening complication requiring intensive care management) complications or recurrent laryngeal nerve injury. Recurrent laryngeal nerve injury is considered when postoperative hoarseness exists and should be confirmed by laryngoscopy, proving paralysis of a vocal cord. The composite outcome measure will be calculated as the number of patients with major morbidity and the number of deceased patients in the first 30 days after the start of treatment. This number divided by the total number of randomised patients will be considered the proportion of patients with major morbidity or 30-day mortality per randomisation group (Table 3).

**Table 3:** Morbidity and 30-day mortality

	Clavien-Dindo classification grade	With mediastinoscopy (***n***=)	Without mediastinoscopy (***n***=)
Endosonography			
*Postoperative complication, No. (%)*			
*Postoperative complication, No. (%)*			
Mediastinoscopy			
*Postoperative complication, No. (%)*			
*Postoperative complication, No. (%)*			
Anatomical lung resection			
*Postoperative complication, No. (%)*			
*Postoperative complication, No. (%)*			
30-day mortality, No. (%)			

*No.* number. Clavien-Dindo classification: grade 1: complication without need for interventions, grade 2: complication requiring pharmacological treatment, grade 3: complication requiring surgical, endoscopic or radiological intervention, grade 4: life-threatening complication requiring intensive care management, grade 5: death

#### Assessment and analysis of secondary outcomes

Patients will be followed during 2 years after start of treatment. A minority of patients will have N2 or N3 disease diagnosed by mediastinoscopy and will therefore possibly be judged ineligible for surgery. In these patients, the start of chemotherapy and/or radiotherapy will be considered as start of follow-up period. Follow-up will be done at 3, 6, 12 and 24 months after start treatment. The hereafter mentioned secondary outcome measures will all be compared among randomisation groups and data will be subsequently presented for the groups.

#### Total number of days of hospital care

The absolute number of days of hospital care in the period from randomisation until 2 years after start of treatment will be registered. Every day in hospital (including outpatient clinic visits and day care treatments) related to NSCLC diagnosis, treatment or follow-up will be included in this outcome measure. Differences between groups will be tested with Mann-Whitney *U* tests.

#### Overall and disease-free 2-year survival

Overall 2-year survival is defined as the proportion of patients alive at 2 years after start of treatment. Disease-free 2-year survival is defined as the proportion of patients alive without evidence of relapse of NSCLC at 2 years after start of treatment. The overall and disease-free 2-year survival will be presented as Kaplan-Meier curves and compared among the randomisation groups using the log-rank test.

#### Generic and disease-specific health related quality of life

**Fig. 2:**
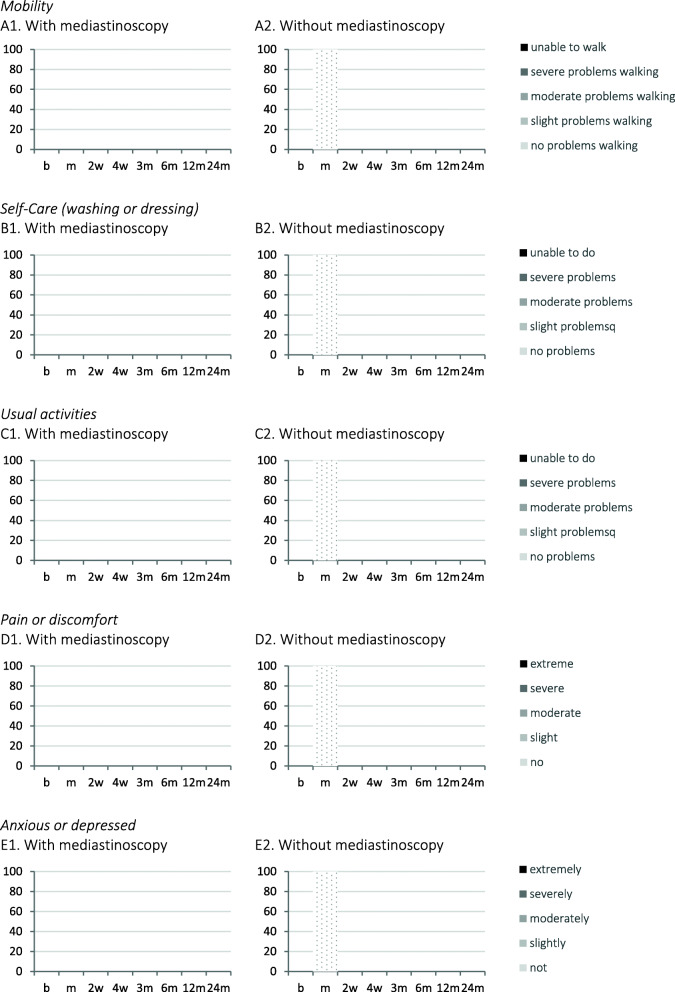
EQ-5D-5L results per domain. Euroqol 5 Dimensions 5 Levels questionnaire. Vertical axis = cumulative percentage; horizontal axis = follow-up moment; b = baseline; m = 1 week after mediastinoscopy; w = weeks after start of treatment; m = months after start of treatment

**Fig. 3:**
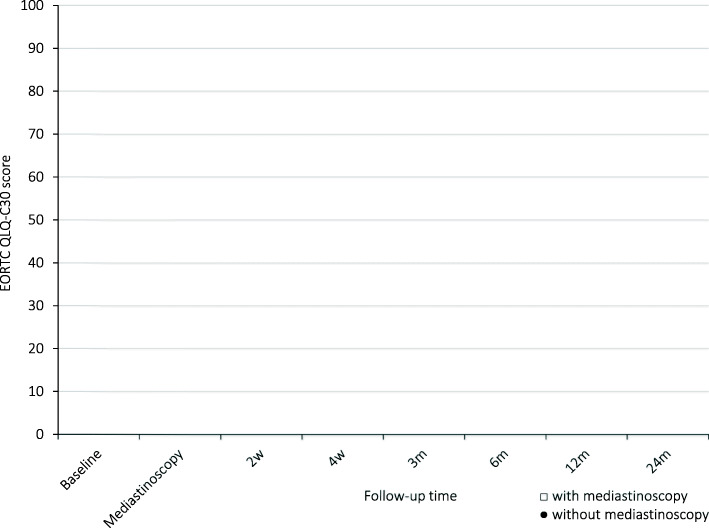
EORTC QLQ-C30 quality of life scores. European Organisation for Research and Treatment of Cancer Quality of life Questionnaire C30. Summary score from 0 to 100, where 100 represents best quality of life. Mean/median score with bars representing standard error/interquartile range. Mediastinoscopy = 1 week after mediastinoscopy; w = weeks after start of treatment; m = months after start of treatment

**Fig. 4:**
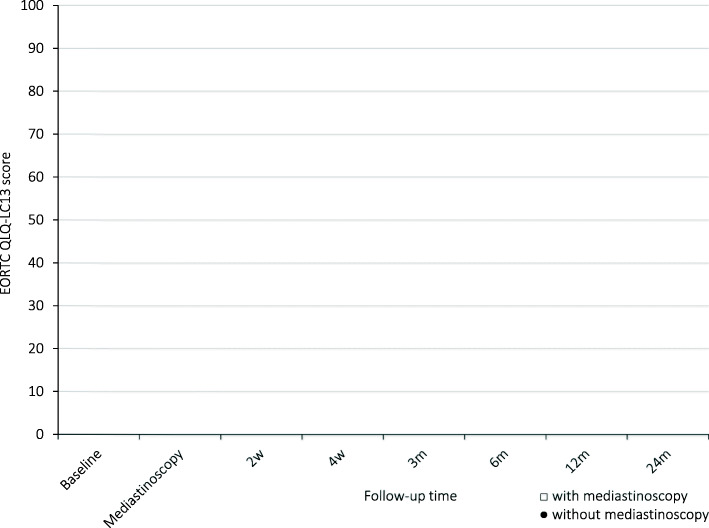
EORTC QLQ-LC13 lung cancer-specific quality of life scores. European Organisation for Research and Treatment of Cancer Quality of life Questionnaire LC13. Score 0–100, 0 = the worst health you can imagine, 100 = the best health you can imagine. Mean/median score with bars representing standard error/interquartile range. Mediastinoscopy = 1 week after mediastinoscopy; w = weeks after start of treatment; m = months after start of treatment

Generic health-related quality of life will be measured using the Euroqol 5 Dimensions 5 Levels questionnaire (EQ-5D-5L) and the European Organization for Research and Treatment of Cancer (EORTC) Quality of life Questionnaire C30 (QLQ-C30). The scoring profiles on the five domains of the EQ-5D-5L (mobility, self-care, activity, pain and anxiety) will be presented in stacked histograms per follow-up moment (Fig. 2). Separately, the Euroqol visual analogue scale representing the quality of life on a scale will be presented (0–100, 0 = the worst health you can imagine, 100 = the best health you can imagine). The EORTC QLQ-C30 incorporates five functional scales (physical, role, cognitive, emotional and social), three symptom scales (fatigue, pain and nausea and vomiting), a global health status and a number of general cancer symptoms (dyspnoea, loss of appetite, insomnia, constipation, diarrhoea and perceived financial impact of the disease). The EORTC QLQ-C30 will provide a summary score from 0 to 100, where 100 represents best quality of life, which will be presented by using a diagram presenting the mean or median score including its standard error or interquartile range per follow-up moment (Fig. 3). The lung cancer-specific quality of life will be measured using the QLQ-LC13 questionnaire, which also provides a summary score from 0 to 100, with 100 representing best quality of life. The results will also be presented in a diagram presenting the scores per follow-up moment (Fig. 4). All quality of life questionnaires will be filled in by the patients at baseline, 1 week after mediastinoscopy (if performed) and after 2 and 4 weeks and 3, 6, 12 and 24 months after start of treatment. Data presentation will be done using figures separately for the questionnaires per randomisation group on all follow-up moments. Comparisons between treatment groups over time will be done using generalised mixed modelling for continuous measures or generalised estimation equations for counts. Absolute values of the quality of life questionnaire results will be reported as tables in supplementary material.

## Discussion

The MEDIASTrial will determine if confirmatory mediastinoscopy can be safely omitted after tumour negative endosonography in invasive mediastinal nodal staging of patients with resectable non-small cell lung cancer. Registration of the study in the Netherlands Trial Register (NL6344/NTR6528) before start of the study, publication of the full study protocol and the present statistical analysis plan before knowledge of any results was done to enhance transparency of scientific behaviour [5]. We expect the inclusion to be complete in 2020 and we aim to publish the primary outcome measure shortly after completion of the inclusion.

## Supplementary Information

**Additional file 1.** Statistical Analysis Plan (SAP) Checklist v 1.02019

**Additional file 2.** Overview of statistical test per outcome

## Data Availability

The datasets and/or analysed data will be available from the principal investigator (FvdB) on reasonable request. The Data Management plan and Trial Master File are managed by the principal investigator (FvdB).

## Abbreviations

NSCLC: Non-small cell lung cancer
SAP: Statistical analysis plan
CONSORT: Consolidated Standards of Reporting Trials
ITT: Intention to treat
PP: Per protocol
WHO: World Health Organization
COPD: Chronic Obstructive Pulmonary Disease
DLCO: Diffusing capacity of the lung for carbon monoxide
FEV1: Forced expiratory volume in the first second of expiration
EBUS-TBNA: Endobronchial ultrasound-guided transbronchial needle aspiration
EUS-FNA: Endoscopic ultrasound-guided fine needle aspiration
ROSE: Rapid on-site evaluation
VATS: Video-assisted thoracoscopic surgery
95% CI: 95% confidence interval
EQ-5D-5L: Euroqol 5 Dimensions 5 Levels questionnaire
EORTC: European Organization for Research and Treatment of Cancer
VAS: Visual analogue scale
