# Supplementary material for: MEDIASTinal staging of non-small cell lung cancer by endobronchial and endoscopic ultrasonography with or without additional surgical mediastinoscopy (MEDIASTrial): a statistical analysis plan
Source: Trials. 2021 Feb 27;22:168. doi: 10.1186/s13063-021-05127-6 (PMC7913384; doi:10.1186/s13063-021-05127-6)
Supplement: Supplementary file 2 — Additional file 2. Overview of statistical test per outcome [file 13063_2021_5127_MOESM2_ESM.docx]

| **Appendix 2.** Overview of statistical test per outcome | | |
| --- | --- | --- |
| **Outcome** | | **Planned statistical test to compare randomisation groups** |
| Baseline characteristics | | None* |
|  | |  |
| Endosonography outcomes | | |
|  | Additional EUS procedures | Mantel-Haenszel chi-squared test or Fisher’s exact test |
|  | Sedation used | Mantel-Haenszel chi-squared test or Fisher’s exact test |
|  | Rapid on site evaluation | Mantel-Haenszel chi-squared test or Fisher’s exact test |
|  | Visualized lymph nodes | Unpaired t-test or Mann-Whitney U-test |
|  | Sampled lymph nodes | Unpaired t-test or Mann-Whitney U-test |
|  | Samples per lymph node | Unpaired t-test or Mann-Whitney U-test |
|  | Patients with cytologically proven N1 | Mantel-Haenszel chi-squared test or Fisher’s exact test |
|  |  |  |
| Cervical videomediastinoscopy outcomes | | None (as results of randomisation only 1 group) |
|  |  |  |
| Surgical reference standard outcomes | |  |
|  | Surgical technique | Mantel-Haenszel chi-squared test or Fisher’s exact test |
|  | Converted operations | Mantel-Haenszel chi-squared test or Fisher’s exact test |
|  | Duration of surgery in minutes | Unpaired t-test or Mann-Whitney U-test |
|  | Type of resection | Mantel-Haenszel chi-squared test or Fisher’s exact test |
|  | Sampled lymph node stations | Unpaired t-test or Mann-Whitney U-test |
|  | Complete lymph node dissections | Mantel-Haenszel chi-squared test or Fisher’s exact test |
|  |  |  |
| Unforeseen N2 disease | | Mantel-Haenszel chi-squared test or Fisher’s exact test |
| Minimal N2 disease | | Mantel-Haenszel chi-squared test or Fisher’s exact test |
|  | |  |
| Major morbidity and 30-day mortality | | Mantel-Haenszel chi-squared test or Fisher’s exact test |
|  | |  |
| Days of hospital care | | Unpaired t-test or Mann-Whitney U-test |
|  | |  |
| Overall and disease free 2-year survival | | Log-rank test (Kaplan Meier estimates) |
|  | |  |
| Quality of life | |  |
|  | EQ-5D-5L | generalized mixed modelling / generalized estimation equations |
|  | EORTC QLQ-C30 | generalized mixed modelling |
|  | EORTC QLQ-LC13 | generalized mixed modelling |
|  | |  |
| 95% confidence interval calculation | | Wilson score interval for proportions |
| Correction for multiple testing | | Benjamini-Hochberg method |
|  | |  |
| *baseline characteristics should be balanced as result of randomisation. Results will only be tested if visual inspection indicates possible significant difference among the randomisation groups.  EQ-5D-5L= Euroqol 5 Dimensions 5 Levels questionnaire; EORTC= European Organization for Research and Treatment of Cancer; QLQ=Quality of Life Questionnaire; C30=cancer specific; LC13=lung cancer specific. | | |
